# The Diagnostic Accuracy of Procalcitonin, Soluble Urokinase-Type Plasminogen Activator Receptors, and C-Reactive Protein in Diagnosing Urinary Tract Infections in the Emergency Department—A Diagnostic Accuracy Study

**DOI:** 10.3390/jcm13061776

**Published:** 2024-03-20

**Authors:** Mathias Amdi Hertz, Isik Somuncu Johansen, Flemming S. Rosenvinge, Claus Lohman Brasen, Eline Sandvig Andersen, Anne Heltborg, Thor Aage Skovsted, Eva Rabing Brix Petersen, Mariana Bichuette Cartuliares, Stig Lønberg Nielsen, Christian Backer Mogensen, Helene Skjøt-Arkil

**Affiliations:** 1 Department of Infectious Diseases, Odense University Hospital, 5000 Odense, Denmark; isik.somuncu.johansen@rsyd.dk (I.S.J.); stig.nielsen@rsyd.dk (S.L.N.); 2 Research Unit of Infectious Diseases, Department of Clinical Research, Faculty of Health Sciences, University of Southern Denmark, 5000 Odense, Denmark; 3 Department of Clinical Microbiology, Odense University Hospital, 5000 Odense, Denmark; 4 Research Unit of Clinical Microbiology, University of Southern Denmark, 5000 Odense, Denmark; 5 Department of Regional Health Research, Faculty of Health Sciences, University of Southern Denmark, 5000 Odense, Denmark; 6 Department of Biochemistry and Immunology, Lillebælt Hospital—Kolding, University Hospital of Southern Denmark, 6000 Kolding, Denmark; eva.rabing.brix.petersen@rsyd.dk; 7 Department of Emergency Medicine, University Hospital of Southern Denmark, 6200 Aabenraa, Denmark; 8 Department of Biochemistry and Immunology, University Hospital of Southern Denmark, 6200 Aabenraa, Denmark

**Keywords:** urinary tract infection, bacteremia, procalcitonin, soluble urokinase-type plasminogen activator receptor, C-reactive protein, inflammatory marker, biomarker, clinical diagnosis

## Abstract

**Background**: Urinary tract infections (UTIs) are a leading bacterial infection in the emergency department (ED). Diagnosing UTIs in the ED can be challenging due to the heterogeneous presentation; therefore, fast and precise tests are needed. We aimed to evaluate the diagnostic precision of procalcitonin (PCT), soluble urokinase plasminogen activator receptors (suPARs), and C-reactive protein (CRP) in diagnosing UTIs, grading the severity of UTIs, and ruling out bacteremia. **Methods**: We recruited adults admitted to three Danish EDs with suspected UTIs. PCT, suPAR, and CRP were used in index tests, while blood cultures, expert panel diagnosis, and severity grading were used in the reference tests. Logistic regression and area under the receiver operator characteristic curves (AUROCs) were utilized to evaluate the models and determine the optimal cut-offs. **Results**: We enrolled 229 patients. PCT diagnosed UTI with an AUROC of 0.612, detected severe disease with an AUROC of 0.712, and ruled out bacteremia with an AUROC of 0.777. SuPAR had AUROCs of 0.480, 0.638, and 0.605, while CRP had AUROCs of 0.599, 0.778, and 0.646. **Conclusions**: The diagnostic performance of PCT, suPAR, or CRP for UTIs or to rule out severe disease was poor. However, PCT can safely rule out bacteremia in clinically relevant numbers in ED patients suspected of UTI.

## 1. Introduction

### 1.1. Background

Urinary tract infections (UTIs) are one of the most common bacterial causes of contact with the emergency department (ED) and one of the most common infections overall [1,2]. In the USA, UTIs account for more than two million ED visits annually and are one of the most common diagnoses for women visiting EDs [3,4]. In Denmark, UTIs lead to the hospitalization of more than 15,000 people annually and are the primary cause of community-acquired bacteremia [5,6].

Despite the high prevalence, UTIs are challenging to diagnose due to a wide range of symptoms and disease severity, varying from mild cystitis to life-threatening septic shock [7,8]. Urine culture-based diagnostic tests are unsuitable for EDs due to the 24–48 h turnaround time. Although urine analysis is faster, it lacks sufficient specificity and has poor positive predictive value [9,10]. This can result in both under- and overdiagnosis of UTIs in the ED, particularly in older patients who often present with vague symptoms [11,12].

In cases of suspected uncomplicated cystitis, it is often safe to delay initiating antibiotics and await diagnostic test results. Conversely, it is vital to initiate antibiotics promptly with a high degree of empirical coverage for suspected pyelonephritis and urosepsis, as these conditions can lead to kidney damage and have a high mortality [13,14]. Thus, we need fast and accurate diagnostic tests in the ED to guide antibiotic prescriptions.

Procalcitonin (PCT), a calcitonin precursor, is an inflammatory marker recognized to have immunoreactive properties [15]. PCT is considered more specific to bacterial infections and is proposed as a marker for severe infection and bacteremia [16,17,18]. The diagnostic accuracy of PCT in diagnosing UTIs in the ED has generally been studied in subpopulations with stringent UTI definitions that may not reflect the clinical diagnosis, and mixed results have been shown [19,20,21].

Soluble urokinase plasminogen activator receptor (suPAR) is the soluble leftovers from the cleavage of the urokinase-type plasminogen activator receptor on the surface of activated immune cells [22]. It has been evaluated as an infection marker, although with varied results [23,24]. Very few published studies have explored the diagnostic value in the ED [25], and only one study in a small cohort of children has evaluated suPAR for diagnosing UTI [26].

C-reactive protein (CRP) is a reliable and widely used inflammation marker and acute phase reactant [27,28]. CRP is sensitive but not specific to infection or any specific infection site [27]. Perhaps due to this well-known lack of specificity, very few studies exist on the diagnostic accuracy of CRP to diagnose UTIs.

Urine cultures are highly specific and sensitive to bacteriuria, but their accuracy in diagnosing UTIs suffers. Sensitivity can be as low as 45%, specificity as low as 72%, and negative predictive value (NPV) as low as 50% [29,30,31]. Despite this, positive urine cultures are often equated to a diagnosis of UTI in the literature, though often supported by the presence of symptoms leading to a selection bias [32].

### 1.2. Aim and Objectives

The aim of this study was to determine the diagnostic value of CRP, PCT, and suPAR in diagnosing UTIs in an ED setting.

The primary objective was to determine the optimal cut-offs and diagnostic precision of CRP, PCT, and suPAR for diagnosing UTI in ED patients with suspected UTI. The secondary objectives were to evaluate the diagnostic precision of these tests in categorizing the severity of UTIs and ruling out bacteremia in patients admitted with a suspected UTI.

## 2. Materials and Methods

### 2.1. Study Design

This is a diagnostic accuracy type 1 multicentre study with prospective data collection, exploring the diagnostic capabilities of three index tests (PCT, suPAR, and CRP) and three reference tests (UTI diagnosis, UTI severity, and bacteremia). This study presents one of the objectives of the multifaceted INDEED (Infectious Diseases in Emergency Departments) study, which aims to develop new diagnostic tools and methods to facilitate rapid and accurate diagnoses, thus reducing the number of non-targeted antibiotic prescriptions in emergency departments [33].

This study was reported according to the STARD statement [34].

### 2.2. Participants

We enrolled patients from the medical EDs of three hospitals in the Region of Southern Denmark: Hospital Sønderjylland in Aabenraa and Sønderborg, Hospital Lillebælt in Kolding, and Odense University Hospital. These three hospitals cover a diverse urban and rural population of 775,000 and are part of Denmark’s tax-funded universal healthcare system.

We consecutively enrolled patients on weekdays and evenings for a year starting 1 March 2021. Six health-care-educated project assistants screened patients admitted to the EDs. If the admitting physician suspected a UTI, the patient was screened for eligibility and invited to participate.

Patients were eligible unless they were below 18 years of age, pregnant, had a severe immunodeficiency, had a prior admittance over 24 h within the last 14 days, tested positive for COVID-19 within the previous 14 days, or if participation would delay life-saving treatment [33].

### 2.3. Blood Sample Procedure

The blood sample for PCT and suPAR analyses was prescribed by the research assistant and taken by a trained laboratory technician or the research assistant in a BD Vacutainer^®^ EDTA tube (BD Switzerland Sarl, Eysins, Switzerland). The collected sample for PCT and suPAR was immediately hand-carried or sent by the TEMPUS600^®^ system (Sarstedt, Nürnbrecht, Germany). It was centrifuged, pipetted, frozen, and stored at −20 °C. The PCT and suPAR samples were thawed only once just before analysis, which was carried out within two months of freezing, and analyzed at the same laboratory.

As part of the standard of care, a trained lab technician took blood samples for CRP analysis in a BD Vacutainer^®^ PST™ Lithium heparin tubes (BD Switzerland Sarl, Eysins, Switzerland), and blood cultures were collected in BioMérieux BACT/ALERT^®^ (BioMerieux, Marcy l’Etoile, France) FA PLUS and FN PLUS culture bottles, if prescribed by the admitting physician.

### 2.4. Tests and Variables

#### 2.4.1. Procalcitonin—Index Test

EDTA plasma was analyzed with BRAHMS Procalcitonin ECL on Cobas^®^ 8000 (Roche Diagnostics GmbH, Mannheim, Germany). Values measured above the upper limit of detection (master curve maximum) > 100 µg/L were set to 100 µg/L, and below the lower limit of quantification, <0.06 µg/L, they were set to 0 µg/L.

#### 2.4.2. Soluble Urokinase-Type Plasminogen Activator Receptor—Index Test

EDTA plasmas were analysed with suPARnostic^®^ TurbiLatex Reagents, (ViroGates A/S, Birkerød, Denmark) on Cobas^®^ 8000, (Roche Diagnostics GmbH, Mannheim, Germany). Values measured above the upper limit of detection, >16 µg/L, were set to 16 µg/L, and below the lower limit of quantification, <1.5 µg/L, they were set to 0 µg/L.

#### 2.4.3. C-Reactive Protein—Index Test

Heparin plasma was analyzed with the C-reactive protein (CRP4) immunoturbidimetric assay (Tina-quant^®^, Roche Diagnostics GmbH, Mannheim, Germany) on Cobas^®^ 8000 (Roche Diagnostics GmbH, Mannheim, Germany). Samples were measured above the lower level of quantification of 0.6 mg/L. The results were collected from the patients’ medical charts and entered into the online data collection tool (REDCap version 10.8.3 to version 12.2.1 by Vanderbilt University, Nashville, TN, USA).

#### 2.4.4. Urinary Tract Infection Diagnosis and Severity—Reference Tests

To avoid the selection bias of using urine cultures as the gold standard, we used a clinical diagnosis made by an expert panel. Two experts out of a panel of 10 experienced emergency and infection medicine specialists independently reviewed each participant’s medical record after at least seven days. Based on medical charts, standard of care laboratory results, microbiology and radiological results, and treatment outcomes, they determined the diagnosis and the severity of the disease in those diagnosed with a UTI. For this study, the diagnoses were dichotomized into UTI and non-UTI.

Severe disease was defined as an expert panel diagnosis of pyelonephritis or urosepsis, while cystitis was defined as mild disease. In case of disagreement, they re-evaluated the medical record and reached a consensus. The experts were blinded to PCT and suPAR but could not be blinded to CRP.

#### 2.4.5. Blood Cultures—Reference Test

Blood culture bottles were carried by hand or car transport to the microbiological department, depending on the site. Culture bottles were incubated for six days or until growth was detected in the BioMérieux BACT/ALERT^®^ VIRTUO^®^ incubation system (BioMerieux, Marcy l’Etoile, France). The result was retrieved from the patients’ charts and recorded in the online data collection tool. The clinical microbiologists were blinded to PCT and suPAR but not CRP.

#### 2.4.6. Other Variables

Age and sex assigned at birth were collected from patients’ charts after obtaining consent and entered into the online data collection tool. During the inclusion process, the research assistants interviewed patients about new symptoms, which they recorded in the data collection tool. Clinical findings from the treating physician were collected from the medical charts.

### 2.5. Statistical Analysis

We used descriptive statistics to summarise the patients’ characteristics. We reported continuous variables as means and interquartile range and categorical as numbers and percentages.

We used univariate logistic regression and area under the receiver operator characteristics curve (AUROC) to evaluate the model and find the optimal cut-off. We used Youden’s index to find the cut-off to diagnose UTI. For severity of disease and bacteremia, we used a 95% sensitivity target as we would use the test to rule out severe disease and bacteremia. Two variables (PCT and CRP) were log converted to fulfill the assumption of linearity unmodified, and the calculations were on the log value and converted back to report the actual cut-off found. We used Pearson residuals to test for the assumption of extreme outliers. We set a ±2 cut-off and recalculated the AUROCs, excluding the extreme outliers, to evaluate their effects. Finally, we calculated the Pearson correlation coefficient between the three markers to evaluate correlation.

Sensitivity, specificity, diagnostic accuracy (DA), negative predictive value (NPV), and positive predictive value (PPV) were calculated from 2 × 2 tables and reported in percentages. The Clopper–Pearson interval was used to calculate the 95% confidence intervals for sensitivity, specificity, and DA, while the standard logit confidence intervals were used for PPV and NPV.

Due to our study design, where patients who were not diagnosed with any infection would be dichotomized into the non-UTI group, it could produce a false association if a test is sensitive to infection but not to UTIs. Therefore, we conducted sensitivity analyses to diagnose UTIs and rule out bacteremia, excluding any patients diagnosed by the expert panel as not having any infection.

The study was powered to show that AUROCs of 0.7 and up were statistically different from 0.5. We estimated that 80% of patients where the admitting clinician suspected UTI had true UTI. We set the significance level to 0.05 (alpha) and the power to 80% (beta). This gave us a minimum sample size of 93.

## 3. Results

### 3.1. Participants

**Figure 1:**
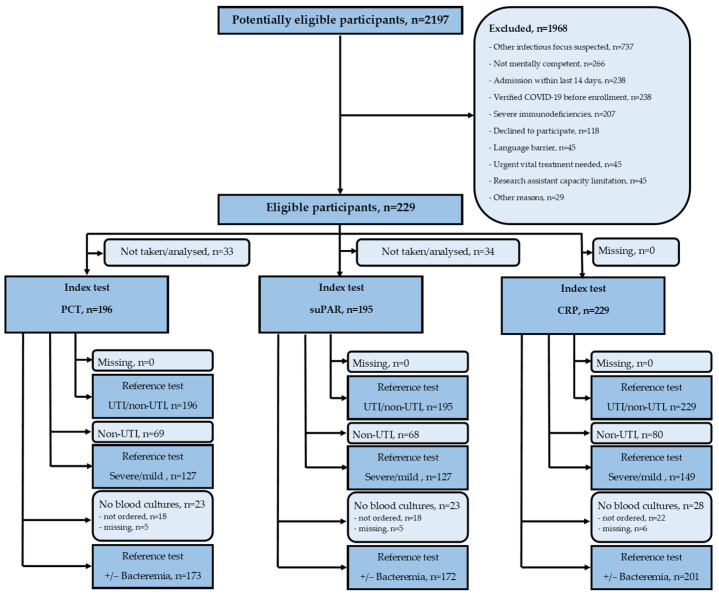
Flowchart of participants, index tests, and reference tests. CRP—C-reactive protein; PCT—procalcitonin; suPAR—soluble urokinase-type plasminogen activator receptor; UTI—urinary tract infection.

We assessed 2197 patients for eligibility, and 1968 (89.6%) were excluded or declined participation. We included 229 (10.4%) patients suspected of having a UTI by the admitting physician (Figure 1). Of these, 149 (65.1%) were retrospectively diagnosed by the expert panel to have a UTI, 45 (19.7%) had other infections, and 35 (15.3%) did not have an infection. All 229 patients had a blood test for CRP; 201 (87.8%) had blood cultures performed, 196 (85.6%) had a PCT, and 195 (85.2%) had a suPAR.

Characteristics of patients with suspected UTI stratified by expert panel diagnosis are presented in Table 1. Patients diagnosed with a UTI had a median age of 75 years, and 59.1% were men. In patients with a UTI, the median PCT, suPAR, and CRP were 0.53 μg/L, 7.2 μg/L, and 136 mg/L, respectively. Of the 149 patients with UTI, 120 (80.5%) had severe disease. Of the 136 with a UTI who had blood cultures taken, 39 (28.7%) had a positive result. Out of 141 patients diagnosed with UTI where complete information was available on symptoms and clinical findings, 106 (75.2%) exhibited classic UTI symptoms (suprapubic or flank pain, increased urinary frequency, dysuria, new urine retention or incontinence, and change of urine appearance or smell). Urine cultures were positive in 74.8% of the 139 UTI patients who had a urine culture taken.

We found a low correlation between the three markers. The Pearson correlation coefficient was 0.31 for PCT and CRP, 0.42 for PCT and suPAR, and 0.35 for suPAR and CRP.

### 3.2. Diagnostic Precision of Procalcitonin

We calculated an AUROC of 0.717 (95% CI 0.642–0.792) for PCT to diagnose UTI in a population of patients suspected of having a UTI. The optimal Youden’s cut-off was calculated to be 0.43 μg/L. When excluding extreme outliers, the model AUROC decreased to 0.648 (95% CI 0.560–0.735). However, the model excluded all values below 0.06, which are biologically plausible; therefore, we do not consider it valid to exclude extreme outliers. In the sensitivity analysis that excluded non-infection patients, the model’s AUROC dropped to 0.612 (95% CI 0.515–0.709) for diagnosing UTI.

For grading the severity of UTI, we found a model AUROC of 0.712 (95% CI 0.590–0.833) and calculated a high-sensitivity cut-off to be 0.08 μg/L.

For ruling out bacteremia in patients admitted with suspicion of UTI, PCT had an AUROC of 0.809 (95% CI 0.737–0.881%). Excluding the non-infected for the sensitivity analysis reduced the AUROC non-significantly to 0.777 (95%CI 0.695–0.858).

See Table 2 for diagnostic values.

### 3.3. Diagnostic Precision of Soluble Urokinase-Type Plasminogen Activator Receptor

The suPAR AUROC was 0.583 (95% CI 0.494–0.671) for diagnosing UTIs, and we found Youden’s cut-off to be 6.5 μg/L. We determined an AUROC for suPAR to grade the severity of UTI to be 0.576 (95% CI 0.438–0.714) and the 95% sensitivity cut-off to be 3.7 μg/L. In ruling out bacteremia, suPAR had a model AUROC of 0.637 (95% CI 0.547–0.727) and a 95% sensitivity cut-off of 4.6 μg/L. See Table 2 for diagnostic values.

### 3.4. Diagnostic Accuracy of C-Reactive Protein

C-reactive protein had a model AUROC for diagnosing UTIs among patients suspected of UTI of 0.723 (95% CI 0.651–0.794) and an optimal cut-off of 71 mg/L. There were no extreme outliers, but when we excluded the non-infected for the analysis, the AUROC was reduced to 0.599 (95% CI 0.503–0.694).

When grading severe disease in UTI patients, we calculated an AUROC of 0.676 (95% CI 0.567–0.785) and a 95% sensitivity cut-off of 19 mg/L.

In ruling out bacteremia in patients suspected of having a UTI, CRP had an AUROC of 0.689 (95% CI 0.598–0.780) with a 95% sensitivity cut-off of 14 mg/L. Excluding extreme outliers, the AUROC increased to 0.782 (95% CI 0.701–0.864), while recalculating without the non-infected reduced the AUROC to 0.646 (95% CI 0.546–0.746). See Table 2 for diagnostic values.

## 4. Discussion

### 4.1. Key Results

PCT demonstrated acceptable discrimination with an AUROC of 0.717 and, with a cut-off value of 0.43 μg/L, a PPV in our population of 83.0% for detecting UTIs. However, the AUROC fell to 0.612 when excluding the non-infected. A cut-off of 0.08 μg/L detected severe disease with a similar performance: AUROC of 0.712, a sensitivity of 88.4%, and a PPV of 85.0. When used to diagnose bacteremia, though, it had a better AUROC of 0.809, and with a 95% sensitivity cut-off of 0.15 μg/L, we found an NPV of 96.3% in our population.

suPAR discriminated poorly in UTI diagnosis and severity grading with AUROCs of 0.583 and 0.576, respectively, and confidence intervals including 0.5. When ruling out bacteremia, it performed slightly better with an AUROC of 0.689, which was significantly different from 0.5, and an NPV of 92.9%.

CRP had an acceptable discrimination of UTI with an AUROC of 0.723, but with an optimal cut-off of 71 mg/L, it gave only a PPV of 77.5% in our population. In grading the severity of UTI and detecting bacteremia, it performed poorly with an AUROC of 0.676 and 0.689, respectively.

### 4.2. Study Limitations

Most studies define UTIs very stringently, almost all requiring a positive urine culture. This excludes the subgroup of patients with a negative urine culture and introduces a risk of including patients with a positive urine culture but no UTI. Our study did not have strict criteria for diagnosing UTI. The diagnosis was based solely on the expert panel’s evaluation. As a result, 52.6% of patients diagnosed with UTI had positive urine cultures and UTI symptoms. In comparison, 20.6% of those not diagnosed with a UTI had a positive urine culture and UTI symptoms. This non-strict definition, in turn, questions the reproducibility and generalization of our results. We tried to ameliorate this by having the experts of different specialties evaluate every patient individually. Since our results are based on a clinical diagnosis, we argue that this study design is more clinically applicable than those with a more stringent UTI definition.

Although the expert panel was blinded to PCT and suPAR, they were not blinded to CRP. This may have affected the accuracy of CRP’s discriminatory performance in diagnosing UTIs and grading severity. The expert panel likely utilized CRP to diagnose, which could have led to an overestimation of its discriminatory performance. Although the microbiologists were not blinded to CRP, their influence is negligible since they were unaware of this study and had no motivation to check CRP levels.

The reference test of the severity of the disease fell outside the power calculations because the prevalence in this group was higher than predicted. A post hoc power calculation with the recorded values found that we had a sufficient number to have enough power to show that an AUC of 0.7 or above is significantly different from 0.5, which is also reflected in the confidence intervals.

Due to our study design, with patients included based on the clinician’s suspicion of UTI after the initial examination, we missed patients with UTI who did not present with typical symptoms. These initially misclassified patients represent an interesting target for further research to optimize treatment and diagnostics.

Some patients did not have blood cultures, PCT, or suPAR taken. Since blood cultures were only obtained if the treating physician prescribed it, we expect patients without blood cultures to have less severe disease and thus a lower risk of bacteremia and a lower prevalence, which would reduce our PPV but increase our NPV. Technical and logistic issues caused missing PCT or suPAR results, which are considered missing completely at random.

### 4.3. Implications for Practice

#### 4.3.1. Procalcitonin

Our study’s AUROC of PCT in diagnosing UTIs matches a similar study that only included women in the ED [20]. The specificity in our study was higher at 78.3% vs. 63%, while our sensitivity was lower at 57.5% vs. 67%. This difference is likely due to their lower cut-off of 0.25 µg/L, as opposed to our higher cut-off of 0.43 µg/L. After testing whether the non-infected patients who ended up in the non-UTI group drove our findings, our AUROC decreased to 0.612, consistent with another similar study [19]. This finding suggests that PCT is not specific to UTIs but only to bacterial infections. Therefore, caution should be exercised when interpreting PCT findings in a clinical setting or designing studies to evaluate the specificity of PCT in a specific infectious focus.

We found an adequate AUROC of 0.712 when using PCT to determine the severity of UTIs using the cut-off of 0.1 µg/L. This analysis does not have the bias mentioned above and is consistent with similar studies with similar cut-offs, which test for the diagnosis of upper versus lower UTIs (AUROC 0.644–0.94) [35]. However, if used in our very high prevalence population, it performs poorly in ruling out severe UTIs. Of the 127 patients with a UTI and a PCT result, only 20 (15.7%) UTIs with severe disease could be ruled out, with an unacceptably high false negative rate (60.0%). Thus, it is inadvisable to use PCT to rule out severe UTI.

If using PCT to rule out bacteremia, however, we calculated a good AUROC 0.809, with a 94.9% sensitivity cut-off of 0.15 µg/L. This aligns with prior studies in similar populations (AUROC 0.72–0.993, cut-offs 0.25 µg/L–3.61 µg/L) [16,36,37,38,39]. Furthermore, when we recalculate without the non-infected, AUROC decreases minimally to 0.777, indicating that this bias does not drive this result. In a population similar to ours and using this cut-off, PCT could rule out bacteremia in 31.2% of patients, giving the clinician the option for narrower or oral antibiotics and earlier discharge from the hospital. Additionally, only 3.7% of patients who test negative for bacteremia will have positive blood cultures, making PCT a viable diagnostic tool in the ED. However, this high sensitivity cut-off should only be used to rule out bacteremia, as it lacks specificity, and 68.9% of the positive PCT tests will have negative blood cultures.

#### 4.3.2. Soluble Urokinase-Type Plasminogen Activator Receptor

We found that suPAR performed poorly in diagnosing UTIs and grading the severity of UTIs with AUROCs, which included 0.5 in the confidence intervals. To our knowledge, no prior studies in adults have evaluated suPAR for UTIs. A similar study found an AUROC of 0.50 for suPAR to predict a bacterial cause of inflammation in sepsis patients [40]. To rule out bacteremia, suPAR has an AUROC that is significantly different from 0.5. In a similar population to ours, if suPAR is used with a cut-off of 4.6 µg/L, it could rule out bacteremia in 16.3% of patients. However, 7.1% of these negative tests will have positive blood cultures. Our results indicate that suPAR has no value as a diagnostic test for diagnosing UTIs and grading the severity of UTIs, and it has minimal value in ruling out bacteremia.

#### 4.3.3. C-Reactive Protein

CRP showed a slightly better, but not statistically significant, AUROC (0.723) compared to PCT in diagnosing UTIs. However, in the sensitivity analysis without non-infected individuals, the AUROC decreased to 0.599. This indicates that the AUROC is primarily influenced by sensitivity to infections alone rather than specific to UTI. To our knowledge, no comparable studies are available. However, a study conducted in nursing homes to diagnose UTIs found that a cut-off of 5 mg/L resulted in a sensitivity of 60.0% and specificity of 50.9%. On the other hand, a meta-analysis evaluating the diagnostic accuracy of CRP in pyelonephritis in children found that with a 20 mg/L cut-off, sensitivity was 94%, and specificity was 39% [41,42]. These results suggest that CRP is not specific enough to diagnose UTIs, and caution should be exercised if using it for that purpose.

Although the AUROC of CRP’s diagnostic capability to grade the severity of disease was acceptable, the NPV was poor. While prior studies have shown a significant association between higher CRP and sepsis, and one study reported an impressive AUROC of 1.0 in diagnosing upper UTIs, our results do not support these findings [18,43,44].

CRP also performed poorly when used to rule out bacteremia. Our AUROC was 0.689, which aligns with previous studies indicating that CRP is a poor diagnostic test for bacteremia in patients with UTI [16,39]. Although we found a sensitivity of 95.7%, we could only rule out 23 patients (11.4%); of those, 2 (8.7%) were false negatives.

## 5. Conclusions

Neither PCT nor CRP can be used as a diagnostic test for UTIs. However, in patients suspected of UTIs in the ED, PCT may be a safe and accurate test to rule out bacteremia, allowing clinicians to prescribe more targeted antibiotics or oral antibiotics and, in some cases, discharge earlier. Our results indicate that suPAR has minimal diagnostic value in UTI patients in the ED.

## Figures and Tables

**Table 1:** Characteristics of patients admitted to the emergency department with suspicion of UTI and stratified by expert panel diagnosis.

Expert Panel Diagnosis	UTI	Non-UTI
no. (%)	149 (65.1%)	80 (34.9%)
Clinical characteristics		
Age, years, median (IQR), n = 229	75 (17)	76.5 (18.5)
Sex, male, no. (%), n = 229	88 (59.1%)	43 (53.8%)
UTI symptoms, no. (%), n = 217	106 (75.2%)	47 (61.8%)
Positive urine culture, no. (%), n = 216	104 (74.8%)	30 (39%)
UTI symptoms & positive urine culture, no. (%), n = 206	70 (52.6%)	15 (20.6%)
Laboratory results		
PCT, median (IQR), μg/L, n = 196	0.53 (2.3)	0.14 (0.36)
suPAR, median (IQR), μg/L, n = 195	7.2 (4.7)	6.3 (5.3)
CRP, median (IQR), mg/L, n = 229	136 (126)	50 (118)
Severity of disease (only UTI), n = 149		
Mild (cystitis), no. (%)	29 (19.5%)	-
Severe (pyelonephritis or urosepsis, no. (%)	120 (80.5%)	-
Blood cultures, n = 201		
Positive, no. (%)	39 (28.7%)	8 (12.3%)
Negative, no. (%)	97 (71.3%)	57 (87.7%)

CRP—C-reactive protein; PCT—procalcitonin; suPAR—soluble urokinase-type plasminogen activator receptor; UTI—urinary tract infection.

**Table 2:** AUROCs, cut-offs, diagnostic values, cross-tabulations, and prevalence for each index test stratified by reference test.

Index Test	Reference Test	n	Model AUROC	EEO AUROC	Non-Inf AUROC	Cut-Off	Sens	Spec	PPV	NPV	DA	TP	FP	FN	TN
PCT	UTI	196	0.717	0.648	0.612	0.43 μg/L	57.5%	78.3%	83.0%	50.0%	64.8%	73	15	54	54
	Severity	127	0.712	0.712	-	0.08 μg/L	95.1%	25.0%	84.5%	54.5%	81.9%	98	18	5	6
	Bacteremia	173	0.809	0.858	0.777	0.15 μg/L	94.9%	38.8%	31.1%	96.3%	51.4%	37	82	2	52
suPAR	UTI	195	0.583	0.581	0.480	6.5 μg/L	66.1%	54.4%	73.0%	46.3%	62.1%	84	31	43	37
	Severity	127	0.576	0.638	-	3.74 μg/L	95.1%	0.0%	80.3%	0.0%	77.2%	98	24	5	0
	Bacteremia	172	0.637	0.679	0.605	4.62 μg/L	94.9%	19.5%	25.7%	92.9%	36.6%	37	107	2	26
CRP	UTI	229	0.723	0.771	0.599	71 mg/L	77.9%	58.8%	77.9%	58.8%	71.2%	116	33	33	47
	Severity	149	0.676	0.778	-	19 mg/L	95.0%	20.7%	83.2%	50.0%	80.5%	114	23	6	6
	Bacteremia	201	0.689	0.782	0.646	14 mg/L	95.7%	13.6%	25.3%	91.3%	32.8%	45	133	2	21

AUROC—area under receiver operating characteristics curve; CRP—C-reactive protein; DA—diagnostic accuracy; FN—false negative, FP—false positive; EEO—excluding extreme outliers; Non-inf—excluding the non-infected patients (only UTI and bacteremia); NPV—negative predictive value; PPV—positive predictive value; PCT—procalcitonin; Sens—sensitivity; Spec—specificity; suPAR—soluble urokinase-type plasminogen activator receptor; TN—true negative; TP—true positive; UTI—urinary tract infection.

## Data Availability

Due to Danish data protection laws, data cannot be made available. The full study protocol is published and freely available [33]. The Supplemental Material is the STARD checklist, required for the STARD criteria.

## Supplementary Materials

The following supporting information can be downloaded at: https://www.mdpi.com/article/10.3390/jcm13061776/s1, STARD 2015 Checklist.

## Abbreviations

AUROC	area under receiver operator characteristics curve
CRP	C-reactive protein
DA	diagnostic accuracy
ED	emergency department
NPV	negative predictive value
PCT	procalcitonin
PPV	positive predictive value
suPAR	soluble urokinase-type plasminogen activator receptor
UTI	urinary tract infection

## References

[1] Foxman B. (2014). Urinary tract infection syndromes: Occurrence, recurrence, bacteriology, risk factors, and disease burden. Infect. Dis. Clin. N. Am..

[2] Schappert S.M., Rechtsteiner E.A. (2011). Ambulatory medical care utilization estimates for 2007. Vital Health Stat. 13.

[3] Foxman B. (2010). The epidemiology of urinary tract infection. Nat. Rev. Urol..

[4] Wolfertz N., Bohm L., Keitel V., Hannappel O., Kumpers P., Bernhard M., Michael M. (2022). Epidemiology, management, and outcome of infection, sepsis, and septic shock in a German emergency department (EpiSEP study). Front. Med..

[5] Danmarks_Statistik Danmarks Statistik, Statistikbanken.dk. https://statistikbanken.dk/ind04.

[6] Pedersen G., Schonheyder H.C., Sorensen H.T. (2003). Source of infection and other factors associated with case fatality in community-acquired bacteremia--a Danish population-based cohort study from 1992 to 1997. Clin. Microbiol. Infect..

[7] Dubbs S.B., Sommerkamp S.K. (2019). Evaluation and Management of Urinary Tract Infection in the Emergency Department. Emerg. Med. Clin. N. Am..

[8] Long B., Koyfman A. (2018). The Emergency Department Diagnosis and Management of Urinary Tract Infection. Emerg. Med. Clin. N. Am..

[9] Chernaya A., Soborg C., Midttun M. (2021). Validity of the urinary dipstick test in the diagnosis of urinary tract infections in adults. Dan. Med. J..

[10] Chu C.M., Lowder J.L. (2018). Diagnosis and treatment of urinary tract infections across age groups. Am. J. Obstet. Gynecol..

[11] Caterino J.M., Stevenson K.B. (2012). Disagreement between emergency physician and inpatient physician diagnosis of infection in older adults admitted from the emergency department. Acad. Emerg. Med..

[12] Gupta A., Petty L., Gandhi T., Flanders S., Hsaiky L., Basu T., Zhang Q., Horowitz J., Masood Z., Chopra V. (2022). Overdiagnosis of urinary tract infection linked to overdiagnosis of pneumonia: A multihospital cohort study. BMJ Qual. Saf..

[13] Evans L., Rhodes A., Alhazzani W., Antonelli M., Coopersmith C.M., French C., Machado F.R., McIntyre L., Ostermann M., Prescott H.C. (2021). Surviving Sepsis Campaign: International Guidelines for Management of Sepsis and Septic Shock 2021. Crit. Care Med..

[14] Gupta K., Hooton T.M., Naber K.G., Wullt B., Colgan R., Miller L.G., Moran G.J., Nicolle L.E., Raz R., Schaeffer A.J. (2011). International clinical practice guidelines for the treatment of acute uncomplicated cystitis and pyelonephritis in women: A 2010 update by the Infectious Diseases Society of America and the European Society for Microbiology and Infectious Diseases. Clin. Infect. Dis..

[15] Deftos L.J., Roos B.A., Parthemore J.G. (1975). Calcium and skeletal metabolism. West. J. Med..

[16] Hoeboer S.H., van der Geest P.J., Nieboer D., Groeneveld A.B. (2015). The diagnostic accuracy of procalcitonin for bacteraemia: A systematic review and meta-analysis. Clin. Microbiol. Infect..

[17] Kapasi A.J., Dittrich S., Gonzalez I.J., Rodwell T.C. (2016). Host Biomarkers for Distinguishing Bacterial from Non-Bacterial Causes of Acute Febrile Illness: A Comprehensive Review. PLoS ONE.

[18] Masajtis-Zagajewska A., Kurnatowska I., Wajdlich M., Nowicki M. (2015). Utility of copeptin and standard inflammatory markers in the diagnostics of upper and lower urinary tract infections. BMC Urol..

[19] Choi J.J., McCarthy M.W., Meltzer K.K., Cornelius-Schecter A., Jabri A., Reshetnyak E., Banerjee S., Westblade L.F., Mehta S., Simon M.S. (2022). The Diagnostic Accuracy Of Procalcitonin for Urinary Tract Infection in Hospitalized Older Adults: A Prospective Study. J. Gen. Intern. Med..

[20] Levine A.R., Tran M., Shepherd J., Naut E. (2018). Utility of initial procalcitonin values to predict urinary tract infection. Am. J. Emerg. Med..

[21] Xu R.Y., Liu H.W., Liu J.L., Dong J.H. (2014). Procalcitonin and C-reactive protein in urinary tract infection diagnosis. BMC Urol..

[22] Thuno M., Macho B., Eugen-Olsen J. (2009). suPAR: The molecular crystal ball. Dis. Markers.

[23] Behrendt N., Ploug M., Patthy L., Houen G., Blasi F., Danø K. (1991). The ligand-binding domain of the cell surface receptor for urokinase-type plasminogen activator. J. Biol. Chem..

[24] Ni W., Han Y., Zhao J., Cui J., Wang K., Wang R., Liu Y. (2016). Serum soluble urokinase-type plasminogen activator receptor as a biological marker of bacterial infection in adults: A systematic review and meta-analysis. Sci. Rep..

[25] Velissaris D., Zareifopoulos N., Karamouzos V., Pierrakos C., Karanikolas M. (2022). Soluble urokinase plasminogen activator receptor (suPAR) in the emergency department: An update. Caspian J. Intern. Med..

[26] Wittenhagen P., Andersen J.B., Hansen A., Lindholm L., Ronne F., Theil J., Tvede M., Eugen-Olsen J. (2011). Plasma soluble urokinase plasminogen activator receptor in children with urinary tract infection. Biomark. Insights.

[27] Gewurz H., Mold C., Siegel J., Fiedel B. (1982). C-reactive protein and the acute phase response. Adv. Intern. Med..

[28] Tillett W.S., Francis T. (1930). Serological Reactions in Pneumonia with a Non-Protein Somatic Fraction of Pneumococcus. J. Exp. Med..

[29] Richards D., Toop L., Chambers S., Fletcher L. (2005). Response to antibiotics of women with symptoms of urinary tract infection but negative dipstick urine test results: Double blind randomised controlled trial. BMJ.

[30] Shallcross L., Gaskell K., Fox-Lewis A., Bergstrom M., Noursadeghi M. (2018). Mismatch between suspected pyelonephritis and microbiological diagnosis: A cohort study from a UK teaching hospital. J. Hosp. Infect..

[31] Vaughn V.M., Gupta A., Petty L.A., Malani A.N., Osterholzer D., Patel P.K., Younas M., Bernstein S.J., Burdick S., Ratz D. (2023). A Statewide Quality Initiative to Reduce Unnecessary Antibiotic Treatment of Asymptomatic Bacteriuria. JAMA Intern. Med..

[32] Bilsen M.P., Jongeneel R.M.H., Schneeberger C., Platteel T.N., van Nieuwkoop C., Mody L., Caterino J.M., Geerlings S.E., Koves B., Wagenlehner F. (2023). Definitions of Urinary Tract Infection in Current Research: A Systematic Review. Open Forum Infect. Dis..

[33] Skjot-Arkil H., Heltborg A., Lorentzen M.H., Cartuliares M.B., Hertz M.A., Graumann O., Rosenvinge F.S., Petersen E.R.B., Ostergaard C., Laursen C.B. (2021). Improved diagnostics of infectious diseases in emergency departments: A protocol of a multifaceted multicentre diagnostic study. BMJ Open.

[34] Bossuyt P.M., Reitsma J.B., Bruns D.E., Gatsonis C.A., Glasziou P.P., Irwig L., Lijmer J.G., Moher D., Rennie D., de Vet H.C. (2015). STARD 2015: An updated list of essential items for reporting diagnostic accuracy studies. BMJ.

[35] Shi J., Zhan Z.S., Zheng Z.S., Zhu X.X., Zhou X.Y., Zhang S.Y. (2023). Correlation of procalcitonin and c-reactive protein levels with pathogen distribution and infection localization in urinary tract infections. Sci. Rep..

[36] Caffarini E.M., DeMott J., Patel G., Lat I. (2017). Determining the Clinical Utility of an Absolute Procalcitonin Value for Predicting a Positive Culture Result. Antimicrob. Agents Chemother..

[37] Ha Y.E., Kang C.I., Wi Y.M., Chung D.R., Kang E.S., Lee N.Y., Song J.H., Peck K.R. (2013). Diagnostic usefulness of procalcitonin as a marker of bacteremia in patients with acute pyelonephritis. Scand. J. Clin. Lab. Investig..

[38] Julian-Jimenez A., Gutierrez-Martin P., Lizcano-Lizcano A., Lopez-Guerrero M.A., Barroso-Manso A., Heredero-Galvez E. (2015). Usefulness of procalcitonin and C-reactive protein for predicting bacteremia in urinary tract infections in the emergency department. Actas Urol. Esp..

[39] Varela-Patino M., Lopez-Izquierdo R., Velayos-Garcia P., Alvarez-Manzanares J., Ramos-Sanchez C., Carbajosa-Rodriguez V., Martin-Rodriguez F., Eiros J.M. (2020). Usefulness of infection biomarkers for diagnosing bacteremia in patients with a sepsis code in the emergency department. Infez. Med..

[40] Kofoed K., Andersen O., Kronborg G., Tvede M., Petersen J., Eugen-Olsen J., Larsen K. (2007). Use of plasma C-reactive protein, procalcitonin, neutrophils, macrophage migration inhibitory factor, soluble urokinase-type plasminogen activator receptor, and soluble triggering receptor expressed on myeloid cells-1 in combination to diagnose infections: A prospective study. Crit. Care.

[41] Latour K., De Lepeleire J., Catry B., Buntinx F. (2022). Nursing home residents with suspected urinary tract infections: A diagnostic accuracy study. BMC Geriatr..

[42] Shaikh N., Borrell J.L., Evron J., Leeflang M.M. (2015). Procalcitonin, C-reactive protein, and erythrocyte sedimentation rate for the diagnosis of acute pyelonephritis in children. Cochrane Database Syst. Rev..

[43] Shigemura K., Tanaka K., Osawa K., Arakawa S., Miyake H., Fujisawa M. (2013). Clinical factors associated with shock in bacteremic UTI. Int. Urol. Nephrol..

[44] Yilmaz G., Koksal I., Karahan S.C., Mentese A. (2011). The diagnostic and prognostic significance of soluble urokinase plasminogen activator receptor in systemic inflammatory response syndrome. Clin. Biochem..

